# The Genotoxic Potential of Organic Emissions from Domestic Boilers Combusting Biomass and Fossil Fuels

**DOI:** 10.3390/toxics13080619

**Published:** 2025-07-25

**Authors:** Jitka Sikorova, Frantisek Hopan, Lenka Kubonova, Jiri Horak, Alena Milcova, Pavel Rossner, Antonin Ambroz, Kamil Krpec, Oleksandr Molchanov, Tana Zavodna

**Affiliations:** 1 Department of Toxicology and Molecular Epidemiology, Institute of Experimental Medicine of the CAS, Videnska 1083, 142 00 Prague, Czech Republic; 2 Centre for Energy and Environmental Technologies, Energy Research Centre, VSB—Technical University of Ostrava, 17. listopadu 2172/15, 708 00 Ostrava, Czech Republic

**Keywords:** domestic heating, particulate matter, polycyclic aromatic hydrocarbons, DNA adducts, 8-oxo-dG, genotoxicity

## Abstract

Solid fuels are still widely used in household heating in Europe and North America. Emissions from boilers are released in proximity to people. Therefore, there is a need to minimise the toxicity of emissions affecting human health to the greatest extent possible. This study compares the genotoxic potential of the emissions of four boilers of modern and old design (automatic, gasification, down-draft, over-fire) operating at reduced output to simulate the real-life combustion fed by various fossil and renewable solid fuels (hard coal, brown coal, brown coal briquettes, wood pellets, wet and dry spruce). Organic emissions were tested for genotoxic potential by analysing bulky DNA adducts and 8-oxo-dG adduct induction. There was no consistent genotoxic pattern among the fuels used within the boilers. Genotoxicity was strongly correlated with polycyclic aromatic hydrocarbon (PAH) content, and even stronger correlation was observed with particulate matter (PM). In all measured variables (PM, PAHs, genotoxicity), the technology of the boilers was a more important factor in determining the genotoxic potential than the fuels burned. The highest levels of both bulky and 8-oxo-dG DNA adducts were induced by organics originating from the over-fire boiler, while the automatic boiler exhibited genotoxic potential that was ~1000- and 100-fold lower, respectively.

## 1. Introduction

Solid fuels are still widely used for household heating, primarily in rural areas worldwide, particularly in cold climate conditions. Fumes originating from heating appliances can contain complete and incomplete combustion products, such as organic compounds and particles. According to the Air Convention under the UNECE (The United Nations Economic Commission for Europe) Convention on Long-range Transboundary Air Pollution [1], the residential, commercial and institutional sectors were responsible for a total PM_2.5_ (particulate matter with aerodynamic diameter smaller than 2.5 µm) emissions of 63% in EU countries and 61% in the Czech Republic in 2021. Recently, there have been plans to reduce the use of fossil fuels. However, biomass, as a renewable energy source, will continue to be used to produce thermal energy in modern combustion devices for household heating, aligning with the new EU climate policy (Fit for 55) [2].

Particle emissions from residential heating depend on (i) the combustion technology, (ii) the conditions for combustion, for instance, air (oxygen) supply and temperature in the combustion chamber, (iii) the type of fuel, (iv) the quality of fuel (moisture, granulometry, and ash content), and (v) the after-treatment technology used [3]. Formed particles carry various mixtures of organic compounds, such as polycyclic aromatic hydrocarbons (PAHs), common toxic products of incomplete combustion [4].

Biomass and fossil fuel combustion in the residential/commercial sector is the most significant source of PAHs related to lung cancer risk. The EPA (Environmental Protection Agency) has designated 16 priority PAHs (listed further) based on their prevalence, persistence, and adverse health effects on humans and other organisms. It has been estimated that globally 60.5% of the produced priority PAHs are an outcome of residential/commercial biomass burning [5]. Several studies highlighted that PM-mediated genotoxicity depends on the PAH content and emission profiles [6,7].

In Europe, household heating air pollution is estimated to be responsible for 61,000 premature deaths each year [8]. A comprehensive meta-analysis found a clear link between PM_10_ and PM_2.5_ concentrations and increased mortality and morbidity caused by cardiovascular diseases, respiratory diseases, and lung cancer. PM_2.5_ showed more consistent associations even at lower exposure levels [9,10].

The design of the combustion appliance used strongly influences the formation of particulate matter (PM) and its toxicity. Old technology appliances produce more particles with more PAHs [11,12,13,14]. Modern appliances produce fewer particles which can be enriched with ash elements [15] or contain higher concentrations of inorganic ions [7].

Operational practices also play an important role in toxicity. Better combustion conditions produce lower particle mass emissions enriched with ash. Intermediate and smouldering combustion situations produced a high amount of particles as well as PAHs [16,17,18] and also induced DNA damage in RAW264.7 cells [17].

Most toxicological studies were focused on biomass combustion, as summarized in meta-analysis of Vicente et al. [19], except for a recent study on by Vicente et al. [20] that focuses exclusively on coal. These studies were carried on a small sample of combustion devices within individual studies, either stove or automatic pellet boilers mainly. Only one study [21] published so far evaluated genotoxicity from both fossil and biomass solid fuel combustion. Currently, we have studied the genotoxic potential of the combustion of various biomass and fossil fuels (hard coal, brown coal, brown coal briquettes, wood pellets, wet and dry spruce). The comparison of biomass and fossil fuels combusted in two modern and two old types of combustion technologies under real combustion conditions (reduced heat output) and further investigation of the particulate matter, gaseous, and particulate PAHs emissions have yet to be described.

Two complementary acellular methods for measuring genotoxicity were selected regarding the emission source: ^32^P-postlabeling and 8-oxo-dG analysis. ^32^P-postlabeling using calf thymus DNA was chosen as the method for measuring genotoxicity due to its specific detection of DNA adducts, which are caused by the significant genotoxic pollutants in boiler emission, such as PAHs, their derivates and heavy metals. DNA adducts are products of covalently bound substances to DNA, and their presence is a risk factor for permanent DNA damage that can lead to mutagenesis and carcinogenesis. The method provides high sensitivity and the ability to detect low levels of DNA adducts. Additionally, 8-oxo-dG analyses were performed to measure oxidative damage, as a marker of the oxidative potential of tested samples. Together, these two methods provide broader insight into genotoxic properties of household boiler emissions, making them a suitable tool for comparative assessment of genotoxic potential and providing valuable data on the genotoxic potential of complex pollutant mixtures.

The aim of this study was to perform a comprehensive comparison of the genotoxic potential of various combustion conditions and to identify their contribution to PAH content and DNA adduct formation. 

## 2. Materials and Methods

We summarize the description of performing combustion tests and using measurement techniques, the analysis of gaseous compounds, organic compounds and particles, the chemical analysis of fuels and the analysis of the genotoxic potential of gaseous and particulate compounds. Our previous study [22] was based on the same combustion tests. The experimental work was conducted with the boilers operating at a reduced output of 40–60%. This is a common practice in household boilers in the Czech Republic, as evidenced by on-site real measurements of modern biomass household boilers [23]. All results are expressed in units related to heat contained in the fuel (units/GJ).

### 2.1. Combustion Tests

**Figure 1:**
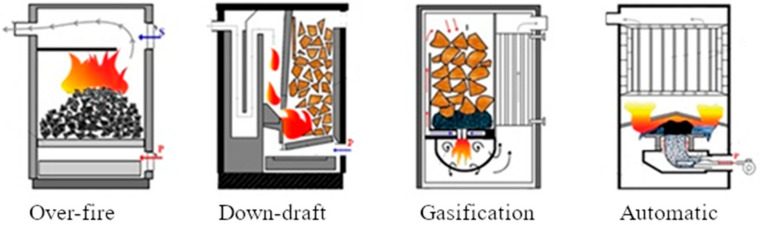
The boiler scheme used—over-fire, down-draft, gasification, automatic (from left to right).

Emission testing was conducted by an accredited testing laboratory. Four different hot-water boiler designs were used in our experiments: modern automatic and gasification boilers and a down-draft boiler with an over-fire boiler representing old technology appliances (Figure 1, Table 1). Three different biomass fuels (dry spruce wood, wet spruce wood, and wood pellets) and three different fossil fuels (hard coal, brown coal, and brown coal briquettes) were combusted depending on the boiler technology. Therefore, we performed sixteen combustion tests (five in the over-fire and down-draft boilers and three in the gasification and automatic boilers). Each combustion test was repeated three times, resulting in forty-eight combustion tests in total.

Table 2 summarizes the combinations of measured fuels with boiler technologies. All tested fuels were combusted with granulometry specified by boiler producers in old-type boiler technologies. The gasification boiler was designed for wood logs and brown coal combustion of specific granulometry (nut 1). The automatic boiler was suitable for the combustion of wood pellets, hard coal, and brown coal of specific granulometry (nut 2).

Dry spruce wood referred to chopped logs with intact bark, typically weighing approximately 0.5 to 1.5 kg each. Wet spruce wood had the same characteristics as dry wood but with a moisture content ranging from 38.0 to 41.6%. Wood pellets were composed of spruce wood sawdust without bark or impurities, featuring a pellet diameter of 6 mm and lengths spanning from 3 to 40 mm according to standards [26]. Brown coal from North Bohemia was utilized for automatic boilers, with a grain size between 10 and 25 mm (nut 2); for other boilers, a grain size of 20 to 40 mm (nut 1) was recommended. Brown coal briquettes were stone-shaped briquettes sized at 25 mm. Hard (black) coal from Poland shared similar grain sizes with brown coal, with nut 2 recommended for automatic boilers and nut 1 for other boiler types.

#### 2.1.1. Measurement Techniques

**Figure 2:**
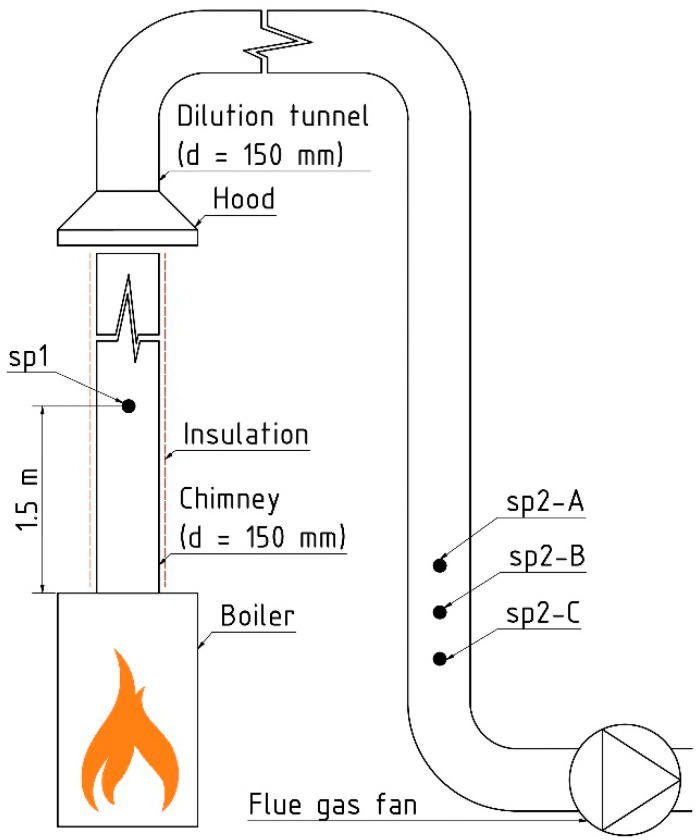
The scheme of the laboratory measuring apparatus. sp1—Sampling point at the boiler outlet—gaseous phase (CO, OGC, O_2_, CO_2_); sp2-A—Sampling point in dilution tunnel—gaseous phase (CO, OGC, O_2_, CO_2_); sp2-B—Sampling point in dilution tunnel—gaseous + particulate phases (PAHs, genotoxicity); sp2-C—Sampling point in dilution tunnel—particulate phase (PM, PM_10_, PM_2.5_, PM_1_, PM_0.1_).

Figure 2 shows the scheme of the laboratory measuring apparatus. Gaseous compounds (CO, organic gaseous compounds—OGC, O_2_, and CO_2_) were measured at site “sp1” behind the boilers. In the dilution tunnel, there were three sampling points: sp2A—gaseous phase (CO, OGC, O_2_, CO_2_), sp2B—gaseous + particulate phases (PAHs, genotoxicity), and sp2C—particulate phase (PM, PM_10_, PM_2.5_, PM_1.0_, PM_0.1_).

The boiler complied with EN 303-5 [25] standards for connection and operation. The dilution tunnel digester was positioned above the chimney according to EPA Method 5G.

Fuel was added after a 2 h stabilisation period to reach 60% of nominal output. However, the actual output was lower (40–60%) due to prolonged fuel burning. The combustion experiment lasted 4.5 h consisting of two periods, and ended with a stable fire bed.

Emissions (O_2_, CO_2_, CO, OGC) were monitored in the chimney (sp1). Aerosol particles were sampled in the dilution tunnel (sp2). O_2_, CO, and CO_2_ levels were also measured in the dilution tunnel (sp2-A) to determine the dilution ratio. This ratio (see Supplementary Materials) was calculated using concentrations at sp1 and sp2-A. Each setting was tested three times for reliability.

#### 2.1.2. Determination of Gaseous Compounds

A paramagnetic analyser (ABB Advance Optima Magnos 16, ABB, Zurich, Switzerland) was applied to determine the concentration of O_2_. A non-dispersive infrared analyser (ABB Advance Optima Uras 14, ABB, Zurich, Switzerland) was connected at sp1 and sp2A to measure the concentrations of CO and CO_2_. The concentration of the organic gaseous compounds (OGC) was determined using a flame ionisation detector (GMS 810, SICK AG, Waldkirch, Germany).

#### 2.1.3. Determination of Organic Compounds

Flue gases for the determination of organic compounds (16 EPA PAHs) were sampled isokinetically in the dilution tunnel according to EN 1948 (filtration–condensation method) [27]. Glass fibre filters were used to collect the dust.

The condensates were extracted in a laboratory shaker using hexane as a solvent. The filters were placed in flasks (50 or 100 mL) with 20 mL of hexane and sonicated for 15 min. The extract was passed through a glass fibre filter into a 100 mL ground-glass flask or glass container. This process was repeated three times. Then, the extract was evaporated in a rotary vacuum evaporator, and a spike of labelled PAHs was added and concentrated in hexane in an amount dependent on the expected concentration. The organic extraction and sample preparation were performed according to EPA 3500c [28]. Further analyses of genotoxic potential were carried out using a complete mixture of both gas phase condensates and extracts from collected particles.

Individual organic components were separated by gas chromatography/mass spectrometry (GC/MS ITQ, Thermo Fisher Scientific, Waltham, MA, USA) into DB-5ms phase (or equivalent) and quantified subsequently by mass spectrometric detection. The determination of PAHs in ambient air using GC/MS was completed according to EPA TO 13A [29].

#### 2.1.4. Analysis of Particles

A 13-stage Dekati^®^ Low Pressure Impactor (DLPI) (Dekati, Tampere, Finland) was applied to determine the mass concentration of individual dust size fractions (sp2-C in Figure 2). The 13 stages allow size-classifying PMs according to the cut-off aerodynamic diameters. A diluter (FPS-4000, Dekati, Tampere, Finland) was inserted into the dilution tunnel, and the 13-stage DLPI was connected behind the diluter throughout the whole combustion test.

A constant suction flow rate of 30 L/min was maintained. Greased aluminium films collected particles. To prevent clogging, the impactor was heated. However, clogging still occurred, requiring additional impactors. Particle loading was limited to 1 mg/stage. Cut-off diameters were calculated based on flow velocity, viscosity, and mean free path. Temperature and pressure within the impactor were monitored while the DLPI was kept at a constant temperature. Collected substrates were weighed using a Mettler Toledo XP6 (Mettler Toledo, Greifensee, Switzerland) microbalance. Horák [30] described in detail the 13-stage DLPI measurement settings and the results of fine and ultrafine particulate matter from these combustion tests.

### 2.2. Chemical Composition of Fuels

The chemical composition (carbon, hydrogen, sulphur, nitrogen, oxygen, water, ash, and net calorific value) of the fuels used is described in the Supplementary Materials [22]. Based on the chemical composition of the fuels, the specific flue gas release from the combusted fuel was calculated (m^3^/kg). Next, fuel consumption was measured during the combustion tests. From these data, the mass concentrations of pollutants were converted into the heat contained in the fuel.

The method for proximate moisture analysis was performed according to standards ČSN 441377 [31] and EN ISO 16948 [32], and for ash according to standards ISO 1171 [33] and EN ISO 18122 [34]. The method for the ultimate analysis of CHN was performed according to standards ISO 29541 [35] and EN ISO 16948 [32], and the analysis of S according to standards ISO 19579 [36] and ISO 16994 [37].

### 2.3. Sample Preparation for an In Vitro Acellular Assay

One millilitre of a mixture of gas phase condensates and extracts from collected particles (see Section 2.1.3) was transferred into DMSO (dimethyl sulfoxide) (Sigma-Aldrich, St. Louis, MO, USA) for DNA treatment. The solvent of the raw extracts was evaporated by nitrogen. Then DMSO was added (0.5–3 mL) according to the weight of the transferred organics to prepare a sample of extractable organic matter (EOM) for genotoxicity testing.

### 2.4. In Vitro Acellular Assay with Bulky DNA Adducts and 8-oxo-dG Analyses

The assay was performed as previously described [38,39]. Briefly, calf thymus DNA (1 mg/mL) (Sigma-Aldrich, St. Louis, MO, USA) was incubated with EOM samples (50 µg EOM/mL) for 24 h at 37 °C with and without metabolic activation of PAHs to DNA reactive metabolites using S9 rat liver microsomal fraction (1 mg protein/mL; Toxila, Pardubice, Czech Republic). Benzo(a)pyrene (1 µM) and DMSO-treated calf thymus DNA samples were used as positive and negative controls, respectively. DNA was isolated by phenol/chloroform/isoamylalcohol extraction and ethanol precipitation, and the samples were kept at −80 °C until further analysis.

^32^P-postlabeling analysis was performed as previously described [40,41]. Briefly, DNA samples (6 µg) were digested by a mixture of micrococcal endonuclease (Sigma-Aldrich, St. Louis, MO, USA) and spleen phosphodiesterase (MP Biomedicals, Strasbourg, France) overnight (17 h) at 37 °C. Nuclease P1 was used for adduct enrichment (Yamasa Corporation, Chiba-ken, Japan). The labelled DNA adducts were resolved by multidirectional thin-layer chromatography on 10 × 10 cm PEI-cellulose plates (Macherey-Nagel GmbH & Co., Dűren, Germany). The solvent systems used for TLC were as follows: D-1: 1 M sodium phosphate, pH 6.8; D-2: 3.8 M lithium formate, 8.5 M urea, pH 3.5; D-3: 0.8 M lithium chloride, 0.5 M Tris, 8.5 M urea, pH 8.0 (all Sigma-Aldrich, St. Louis, MO, USA). Autoradiography was carried out at −80 °C for 6–24 h. The radioactivity of distinct adduct spots and diagonal radioactive zones was measured by liquid scintillation counting. To determine the exact amount of DNA in each sample, aliquots of the DNA enzymatic digest (1 µg of DNA hydrolysate) were analysed for nucleotide content by reverse-phase HPLC with UV detection, which in addition simultaneously allowed for controlling the purity of DNA. DNA adduct levels were expressed as adducts per 10^8^ nucleotides and then recalculated to units related to heat contained in the fuel (relative units/GJ). A B(a)P-diol-epoxide-DNA adduct standard was run in duplicates in each post-labelling experiment to control for inter-assay variability.

8-oxo-dG levels, a marker of oxidative DNA damage, were determined in EOM treated (50 ug EOM/mL) DNA samples using competitive ELISA performed according to a modified protocol [42]. The assay was run in three independent experiments; mean 8-oxo-dG levels per 10^6^ guanosine molecules, and standard deviations were calculated and used for statistical analyses.

### 2.5. Statistical Analysis

Statistical analysis was performed using R statistical software (Version 4.4.2). ANOVA with post hoc Tukey HSD was used for boilers and fuels comparisons when the stability of the variance assumption was met (Bartlett’s Test). Otherwise, a non-parametric test was utilised (Kruskal-Wallis test, post-hoc Dunn test), as indicated in the text. For testing brown coal briquette among boilers, a *t*-test was used. The level of significance was chosen at 0.05.

In total, forty-eight combustion tests were performed, and each combustion test was repeated three times, providing enough data for statistical analysis.

## 3. Results

### 3.1. Particulate Matter and PAH Content

**Figure 3:**
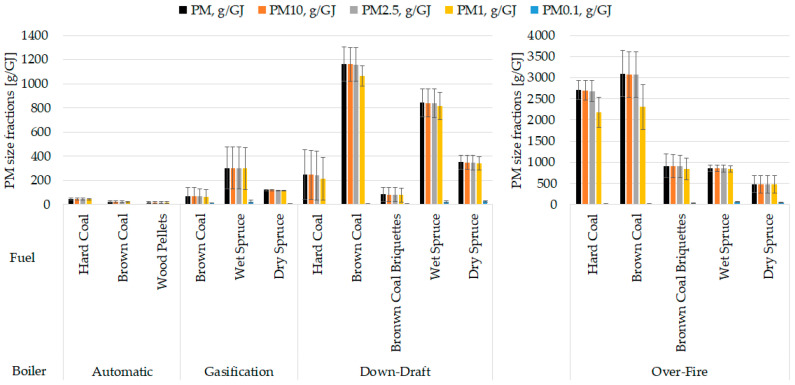
Comparisons of mass proportions of particle size fractions. The results are expressed as mean (n = 3), error bars indicate standard deviation. Statistical significances are listed in Supplementary Materials in Table S4.

As shown in Figure 3, a significant proportion of the particle mass was in the PM_1_ size fraction for all boiler types. The finest fraction PM_0.1_ was also noticeable (0.5–13%) despite its low weight (the weight is directly proportional to the third power of the particle diameter). The produced particle masses differed greatly among boilers. Modern boilers significantly reduced the emission of particles compared to old technology boilers, as expressed in PM mass per fuel energy. For instance, particulate matter from fossil fuels was reduced by 98.3% (hard coal) and 99.3% (brown coal) in automatic boiler vs. over-fire boiler, while PM from biomass was reduced by 75.5% (dry wood) and by 64.5% (wet wood) in gasification boiler vs. over-fire boiler (see Table 3; for more detail, see Supplementary Materials). Brown coal and wet spruce showed statistically significant differences between automatic and over-fire boilers; old boilers emitted more PM (all *p*-values and measured average values are shown in Supplementary Materials Table S4).

Diverse fuels were burned in the boilers, as the boiler designs are incompatible with all the selected fuels. However, every boiler was filled with brown coal and at least one biofuel (wet spruce, dry spruce, wood pellets). Although wet spruce produced consistently more particles than dry spruce, there was only a statistically significant difference in the down-draft boiler (Figure 3).

Fossil fuels were represented by hard and brown coals and brown coal briquettes. No clear pattern, was observed among burning fossil or biofuels within the boilers, as evaluated from *p*-values. Several significant differences among fuels are shown in the Supplementary Materials.

**Figure 4:**
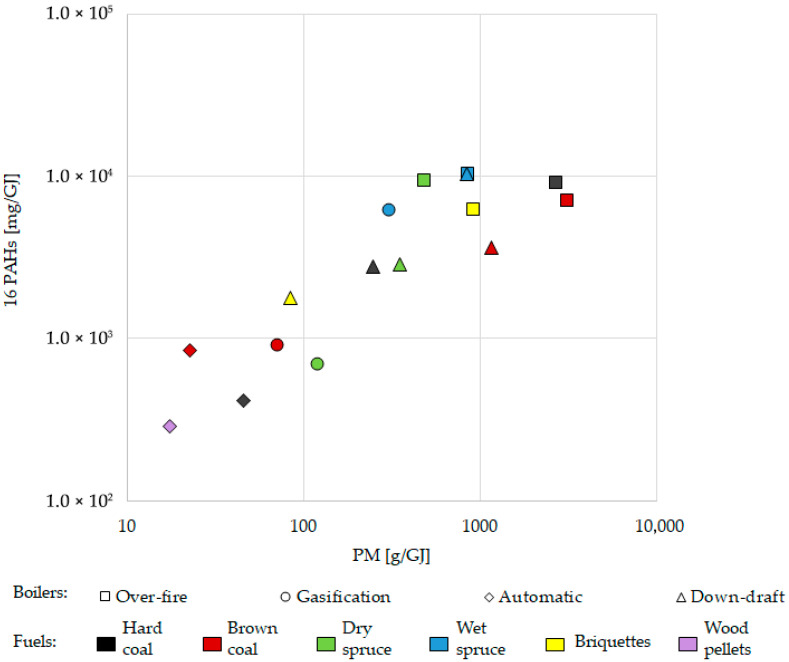
Correlation of the content of 16 total polycyclic aromatic hydrocarbons (PAHs) with particulate matter (PM) in the emissions from different boilers using different fuels. Results are related to heat contained in the fuel (GJ). Wet spruce generated the highest PAH levels within all the boilers (Figure 5); however, a significant statistical difference was not observed (see Table S3, Supplementary Materials for more detail). Spearman’s rank correlation *p*-value < 0.001, ρ = 0.824.

Sixteen priority PAHs (namely naphthalene, acenaphthylene, acenaphthene, fluorene, phenanthrene, anthracene, fluoranthene, pyrene, benzo[a]anthracene, chrysene, benzo[b]fluoranthene, benzo[k]fluoranthene, benzo[a]pyrene, benzo[g,h,i]perylene, indeno [1,2,3-c,d]pyrene, and dibenz[a,h]anthracene) in gaseous and particulate phases were analysed. The correlation in Figure 4 shows that higher PM emissions were associated with higher emissions of 16 PAHs (Spearman’s rank correlation *p*-value < 0.001, ρ = 0.824). This correlates with to the nature of these emissions, which are the products of incomplete combustion. As described for particulate matter, modern boilers reduced the emission of PAHs compared to old technology boilers, as expressed in PAHs mass per fuel energy (mg/GJ) except for wet spruce in the gasification boiler. It is assumed that wet wood would produce more PAHs than dry wood. The calorific value of wet wood is lower than that of dry wood, and the combustion efficiency decreases with increasing moisture content (the total water content in wet spruce was 38–42%).

**Figure 5:**
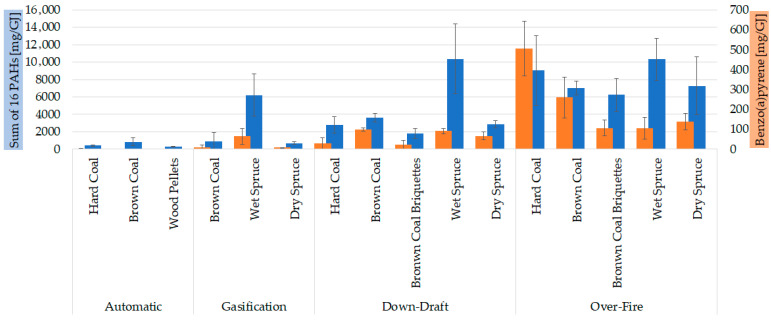
Comparison of 16 EPA priority PAHs with B[a]P for different boilers and fuels at reduced output. The results are expressed as mean (n = 3); error bars indicate standard deviation. Statistical significances are listed in Supplementary Materials in Table S4.

The CHMI (Czech Hydrometeorological Institute) measures and evaluates air pollution; the most important pollutants adversely affecting human health, such as benzo(a)pyrene (B[a]P), have ambient air limits [43]. Therefore, we added the emission of B[a]P in Figure 5 as an important parameter for PAHs emissions. Herein, it is confirmed that the reduction in B[a]P is more than 99% due to the better combustion in the automatic boiler than in the old combustion technology (over-fire boiler) [44]. It is apparent from Figure 5 that the over-fire boiler had the highest 16 PAHs emission and B[a]P emission from coal combustion. However, B[a]P emission was comparable to other combustion technologies, except for the automatic boiler, which had the lowest PAHs emissions. This could be addressed to the fact that other PAHs and organics were produced more from over-fire boilers, which had the worst combustion conditions. Interestingly, the combustion of wet spruce had a similar pattern in all boilers (except for automatic boilers where wood pellets were combusted).

### 3.2. Genotoxic Potential

#### 3.2.1. DNA Adducts

**Figure 6:**
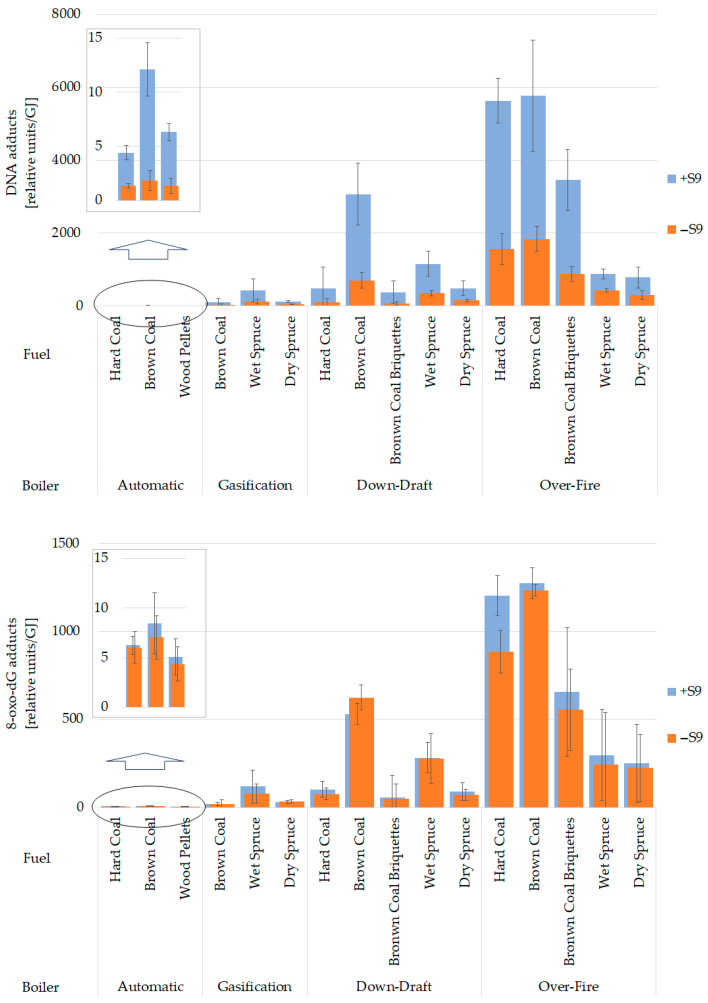
Total levels of bulky DNA adducts (**upper graph**) and 8-oxo-dG DNA adducts (**lower graph**), and the differences between emission samples with (+S9) and without metabolic activation (−S9). The results are expressed as mean (n = 3) adduct levels per 10^8^ nucleotides and 10^6^ guanosines for bulky and 8-oxo-dG DNA adducts, respectively, related to heat contained in the fuel (unit/GJ). Statistical significances are listed in Supplementary Materials Table S4.

**Figure 7:**
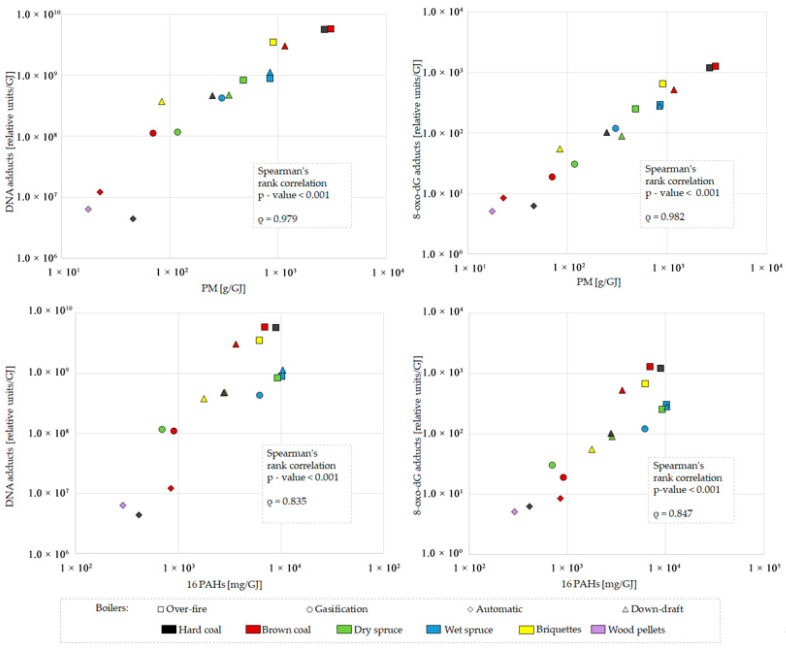
Correlation of bulky DNA adducts (**left graphs**) and 8-oxo-dG DNA adducts (**right graphs**) generated by metabolically activated (+S9) emission samples from different boilers using different fuels with particulate matter (PM) (**top graphs**) and 16 priority polycyclic aromatic hydrocarbons (PAHs) (**bottom graphs**). Results are related to heat contained in the fuel t (GJ).

The highest levels of bulky DNA adducts were induced by organics originating from the over-fire boiler, while the automatic boiler exhibited ~1000-fold lower genotoxic potential (Figure 6, top graph). DNA adduct formation correlates with PM mass (Figure 7, top left graph) for samples with metabolic activation by rat liver microsomal S9 fraction (Spearman’s rank correlation *p*-value < 0.001, ρ = 0.979). This correlation was more substantial than with the sum of 16 priority PAHs (*p*-value < 0.001, ρ = 0.835; Figure 7, bottom left graph). As both variates behaved similarly, the induction of DNA adducts by emitted organic compounds followed the pattern of PM emission. As shown in the top graph in Figure 6, DNA adducts were more frequent in DNA treated by emissions produced by older boilers than by new technology boilers. This tendency was confirmed between automatic and over-fire boilers, and between gasification and old boilers when dry spruce was burned. Much higher total DNA adduct levels in emission samples activated by S9 fraction compared to samples without metabolic activation suggest a substantial contribution of genotoxic PAHs to adduct formation (Figure 6, top graph).

The fuels affected DNA adduct induction in dependence on the boiler type. Emissions from the down-draft and automatic boilers induced significantly more adducts if brown coal was burned. Over-fire and gasification boilers did not show any significance. All *p*-values and measured average values are shown in Supplementary Materials. Overall, the emissions obtained from the automatic boiler were orders of magnitude lower than those of all the other boilers (Figure 6, top graph), while older boilers caused greater genotoxic damage.

#### 3.2.2. Oxidative DNA Damage

The bottom graph in Figure 6 shows that the highest levels of 8-oxo-dG were induced by organic compounds originating from the over-fire boiler. In contrast, the automatic boiler emissions resulted in the lowest observed genotoxicity. Similar to the formation of DNA adducts, 8-oxo-dG levels were correlated with PM (for S9 activated samples, Spearman’s rank correlation *p*-value < 0.001, ρ = 0.982) (Figure 7, top right graph) and to a lesser extent also with the sum of 16 priority PAHs (*p*-value < 0.001, ρ = 0.847) (Figure 7, bottom right graph).

Figure 6 further illustrates that the differences between +S9 and −S9 samples in 8-oxo-dG levels are much lower than those for bulky DNA adducts generated mainly by activated PAHs. This finding suggests that metabolic activation is not required for the formation of 8-oxo-dG by emission components.

Significant differences existed between new and old technologies in brown coal combustion in 8-oxo-dG levels (Figure 6, bottom graph). Other results did not follow clear pattern, as evaluated by *p*-values. All statistical results and measured average values are shown in the Supplementary Materials.

8-oxo-dG adducts produced by different fuels copied, to some extent, the result from the DNA adducts (Figure 6). Emissions from the down-draft boiler induced significantly more 8-oxo-dG adducts if brown coal was burned. Additionally, brown coal briquettes induced considerably lower levels of 8-oxo-dG than wet spruce in the over-fire boiler. Over-fire boiler 8-oxo-dG adducts showed differences in black and brown coals compared with biofuels, while biofuels reduced oxidative damage to DNA. Results from gasification and automatic boilers did not reveal any significant differences in 8-oxo-dG adduct formation among fuels (Figure 6, bottom graph).

## 4. Discussion

We studied four household boilers for solid fuels running on commonly used fossil and renewable fuels. Particle and gas emissions and their genotoxicity were examined. Most exhaust particles were smaller than 1 µm (PM_1_), i.e., in a dimension that is particularly relevant for pulmonary uptake. Due to a combination of persistence in the atmosphere [45] and the ability to reach and deposit deeply in the lungs [46], PM_1_ represents a higher risk for human health than larger particles. PM_1_ was more dangerous for children’s health than PM_2.5_ [47,48]. Household boilers are often operated manually; this also means that emissions from the appliance can enter indoor spaces directly when the operator opens the chamber. Furthermore, emissions from combustion are released into the surrounding air. The impact of PM exposure on cardiovascular, respiratory diseases and cancer is well documented [9,10,49]. However, PM has also had an impact on health aspects emerging recently. Metanalysis revealed a positive association between maternal exposure to PM_2.5_ and autism spectrum disorder in their children [50]. Furthermore, a link was also found between chronic exposure to PM_2.5_ and the risk of type 2 diabetes [51].

This study operates with units relative to the energy obtained from the burning fuel. The amount of emitted PM was correlated to a certain extent with emitted PAHs and the genotoxicity of combined extracted organics with the gas phase condensate. The more particles were produced, the more substantial the genotoxic effects were observed. Most of the studies are in concordance with lowering toxicity by new technology boilers which lead to better combustion and fewer products of incomplete combustion [7,11,12,14,52,53,54]. Considering this fact, there is a rationale for programs supporting the replacement of old boilers with new technologies.

Arif et al. [55] concluded that the particle-bound PAHs in PM_0.4–1_ from softwood and beech wood chips combustion were most likely the cause of toxic effects in human lung cells compared to heavy metals. Additionally, PAHs adsorbed on the particle surface might be more accessible to cells than gaseous PAHs, as the particles could deliver PAHs deep into the lungs and inside the cells by particle uptake [56]. In our study, both PM and PAHs were strongly correlated with the genotoxicities of organic extracts. However, correlations were more substantial for PM, which indicates that other organics than detected PAHs are responsible for an important part of the genotoxicities of the organic extracts combined with gas condensates. It is likely that a large part of the remaining genotoxicity can be attributed to PAH derivatives (nitro-PAHs and oxo-PAHs), which are formed along with PAHs during combustion [57,58]. Some of these derived PAHs are much more toxic than their parent forms [59,60,61,62]. Oxo-PAHs and nitro-PAHs were produced in large quantities within reduced output [63] which corresponds to our experimental condition. Libalova et al. [64] demonstrated that higher genotoxicity of neat gasoline emission was caused by increased levels of nitro-PAHs in organic extract in comparison with gasoline blended with ethanol. Other inorganic ions, metals or matter such as soot/carbon core would contribute to overall toxicity of emissions; however, this study focuses exclusively on the organic part of the emission, and the inorganics were removed by the extraction process.

The impact of combustion unit construction on emission reduction is more significant than that of fuel. Fuel quality is another crucial factor determining the quantity of emissions and genotoxicity (e.g., moisture content, fuel granulometry). When combusting wet wood compared to combusting dry wood, more PM and PAH emissions were produced, which were related to higher genotoxicity. A higher moisture content associated with the completeness of combustion can lead to higher toxic potential [55]. Kasurinen et al. [53] reported that log wood combustion is considerably more harmful than wood pellet combustion. We agree; however, the influence of combustion technology should also be considered in our case. The difference in genotoxicity between hardwood and softwood has yet to be studied.

In vitro toxicity of particulate emissions from residential biomass combustion was described in detail in the latest overview [19]. Indoor PM from residential coal combustion and its toxicity were described in the study [20]. The combustion conditions representing efficient, intermediate and smouldering situations were characterised by Uski et al. [17], who showed that samples from less efficient combustion evoked more significant DNA damage [12]. Furthermore, there are differences in emissions even within a burning cycle when an ignition phase emits a higher amount of PM and PAHs [65]. Manually operated boilers should be operated preferentially at nominal output, producing significantly less pollutants than at reduced output [66]. This is most easily achieved by installing a heat storage tank in the heating system. However, most manually operated combustion appliances in the Czech Republic and Poland are not installed with heat storage tanks. Therefore, they are mainly operated at reduced output for most of the heating season. Particle emissions per energy unit produced from efficient combustion conditions were considerably lower than from smouldering combustion conditions. We focused on organic species (PAHs) influencing the genotoxic potential; however, inorganic ash species should also be considered in future studies. As Leskinen et al. [16] presented, efficient combustion conditions emitted fine particles with the highest potential for cell death due to their composition (inorganic ash species). Niu et al. [67] also identified organic and elemental carbon, water-soluble ions, and high-molecular-weight PAHs as crucial chemical components responsible for cell oxidative and inflammatory responses.

The difference in genotoxicity was observed even in the modern automatic boiler, where emissions from brown coal were significantly more genotoxic than those from other fuels. Although this difference is significant, it is unimportant because genotoxicity is still much lower than emissions from older boilers. However, the brown coal showed the highest genotoxicities in old down-draft boiler, too, when the level of genotoxicity was not negligible. A study of Ihantola et al. [21] did not show any significant difference in genotoxicity between brown coal briquettes and spruce. Combustion tests were carried out on a non-heat-retaining chimney stove, which is most closely comparable to the over-fire boiler, which either did not show any statistically significant differences in DNA adducts between brown coal and other fuels. However, there were differences in oxidative damage of DNA when hard and brown coals caused higher oxidative DNA damage than spruce (statistical significances are listed in Supplementary Materials in Table S4). A higher content of sulphur and nitrogen in the coal compared to wood or other biofuels leads to higher emissions of sulphur dioxide and nitrogen oxides, molecules with adverse effects on human health and the environment. Coal is often enriched by toxic elements (such as fluorine, arsenic, selenium, mercury, and lead) that can be released into the air by combustion [8,68]. Differences among coals, their emissions and oxidative potentials were previously demonstrated [69]. Coal contains condensed aromatic hydrocarbons and inherent PAHs, which evaporate during combustion. A study by Han et al. [70] found that coal ignition at a lower temperature (500 °C) produced two orders of magnitude more PAHs than combustion at a higher ignition temperature (800 °C). Evaporated PAHs were pyrolyzed at a higher temperature, which led to a substantial decrease in PAH mass. Currently, fossil fuels in residential combustion devices are still used in the central and eastern parts of Europe and comprise a negligible amount of fuel combusted in households.

About 45.1% of fossil fuels (brown coal, briquettes, hard coal, and coke) and about 66.9% of renewable fuels (firewood, wood briquettes, wood pellets, vegetable and agro-fuels) were used in 2020 by households in the Czech Republic from total energy consumption [71].

In 2020, households in the Czech Republic used approximately 45.1% of fossil fuels (brown coal, briquettes, hard coal, and coke) and about 66.9% of renewable fuels (firewood, wood briquettes, wood pellets, and vegetable and agro-fuels) for their total energy consumption [71].

A limitation of the study concerns the use of the acellular genotoxicity testing by the ^32^postlabeling method which cannot reflect he wide spectrum of genotoxic and mitigatory processes in a living organism. On the other hand, this simple acellular method enabling consistent and sensitive detection of a defined genotoxic mechanism across a broad range of emission samples, without interference from cellular defence mechanisms, is advantageous for screening purposes, as in vivo systems could mitigate or modulate the initial genotoxic insult through anti-inflammatory and antioxidant enzymatic responses, potentially masking the intrinsic genotoxic potential of the pollutants being evaluated. This simple approach is thus suitable as a preliminary tool for the selection of combustion conditions for more detailed and realistic investigation of genotoxicity covering wider spectrum of genotoxic effects in advanced air-liquid exposure cellular models for the future studies.

Another limitation of this study is the higher variability of the data, especially in manually operated boilers for solid fuel combustion. This variability is primarily associated with the loading of wood logs or lump coal in the combustion chamber and the nature of the combustion process through the fuel layer. The complex of chemical processes that occurs in the fuel layer includes drying, pyrolysis (devolatilization), and char/coke formation. During the combustion experiments, we followed the EN 303-5 standard [25], which describes and recommends the procedure and settings for combustion devices. We are aware that a higher number of combustion tests would enhance the statistical robustness and reliability of the data; however, we consider the overall conclusions of the study to be valid. Specifically, the observed differences in emissions and genotoxicity between modern automatic boilers and manually operated boilers are substantial, spanning several orders of magnitude, and would not be fundamentally altered by additional data collection.

## 5. Conclusions

Our study demonstrates that boiler technology plays a more critical role in determining emissions and the genotoxicity of their organic components than the type of fuel used. Significant differences in genotoxicity were observed between older and modern boiler technologies, even when the same fuel was burned. Automatic boiler technology produced the lowest amount of the measured pollutants (PM and PAHs) and reduced these emissions by more than 98% in comparison with the over-fire boiler. Related to this, the automatic boiler exhibited the lowest genotoxic potential. This indicates that advancements in boiler design can effectively reduce toxic emissions. No consistent genotoxicity patterns were observed among different fuels within each boiler type, supporting the dominant influence of the boiler technology. The stronger correlation of genotoxicity with PM emissions compared to PAH content in EOM suggests that other substances in the emissions contribute significantly to the genotoxic potential. These findings highlight the importance of modernising boiler technology to mitigate the health risks associated with household heating emissions. It is recommended to combust the fuels for which the boilers were designed with special granulometry and optimal moisture. Overall, certified wood pellets in the automatic boiler released the lowest gaseous and particulate emissions.

In the Czech Republic, the replacement of old boilers with modern ones is financially supported (SFŽP ČR, 2024). In our study, we present not only emission factor values but also the genotoxic potential of organic compounds such as PAHs. The health risks posed to inhabitants by gaseous and particulate pollutants from domestic heating sources can thus be more effectively communicated to policy makers.

## Figures and Tables

**Table 1:** The information about boilers used—overfire, down-draft, gasification, and automatic.

Boiler	Over-Fire	Down-Draft	Gasification	Automatic
Boiler Type	Older, typical in Eastern Europe	Older, typical in Eastern Europe	Modern	Modern
Fuel Transport	Manual	Manual	Manual	Automatic
Reference fuel	Wood, Coke, Hard Coal	Brown Coal	Brown Coal, Wood	Brown Coal, Wood pellets
Nominal heat output	22.5 kW (wood), 30 kW (coke)	32 kW	20 kW	25 kW
Primary air	Primary air is directed through the grate to the fuel layer.	Primary air flows through the grate to the fuel.	Primary air is directed into the fuel chamber to initiate the gasification process.	Combustion air is introduced into the combustion zone through the cast-iron burner, partly flowing directly through the fuel layer in the middle part of the burner, and partly through the slits in the burner where the fuel rests on the upper part.
Secondary air	Secondary air enters above the combustion chamber and heat exchanger.	Secondary air inlets are positioned on both the left and right sides of the boiler. The secondary air enters the flames in the initial part of the heat exchanger.	Secondary air is injected into the wood gas through nozzles between the fuel and combustion chambers.	Due to the integrated design, distinguishing between primary and secondary combustion air is difficult.
Emissions	Class 1 (EN 303-5:1999) [24]	Class 2 (EN 303-5:1999) [24]	Class 3 (EN 303-5:2021) [25]	Class 5 (EN 303-5:2021) [25]

**Table 2:** Combinations of measured fuels with boiler technologies, granulometry (mm) or mass of wood log (kg) and moisture (wt%).

Type of Boiler	Origin of Fuel	Type of Fuel	Granulometry/Mass of Wood Log	Moisture (%)
Over-fire boiler	Fossil fuel	Hard coal (nut 1)	20 to 40 mm	4.9
		Brown coal (nut 1)	20 to 40 mm	29.4
		Brown coal briquettes	25 mm	18.6
	Biomass	Dry spruce logs	0.5–1.5 kg	12.2
		Wet spruce logs	0.5–1.5 kg	41.6
Down-draft boiler	Fossil fuel	Hard coal (nut 1)	20 to 40 mm	4.9
		Brown coal (nut 1)	20 to 40 mm	29.4
		Brown coal briquettes	25 mm	18.6
	Biomass	Dry spruce logs	0.5–1.5 kg	8.5
		Wet spruce logs	0.5–1.5 kg	38.0
Gasification boiler	Fossil fuel	Brown coal (nut 1)	20 to 40 mm	29.4
	Biomass	Dry spruce logs	0.5–1.5 kg	8.5
		Wet spruce logs	0.5–1.5 kg	40.1
Automatic boiler	Fossil fuel	Hard coal (nut 2)	10 to 25 mm	11.5
		Brown coal (nut 2)	10 to 25 mm	18.7
	Biomass	Wood pellets	6 mm (diameter), 3–40 mm (length)	6.0

**Table 3:** Mean and standard deviation (sd) of total suspended particles (TSP) and 16 PAHs.

		TSP, g/GJ		16PAHs, mg/GJ	
Boiler	Fuel	Mean	sd	Mean	sd
Automatic	Hard coal	45.7	6.2	411.6	63.0
	Brown coal	22.6	8.5	850.5	459.4
	Wood pellets	17.6	9.9	288.8	42.8
Gasification	Brown coal	70.8	70.3	913.5	1021.2
	Wet spruce	303.4	174.4	6218.1	2404.8
	Dry spruce	118.7	3.6	702.3	171.0
Down-draft	Hard coal	247.4	204.1	2780.0	942.1
	Brown coal	1162.5	141.6	3625.5	499.6
	Brown coal briquettes	84.0	57.5	1783.9	559.7
	Wet spruce	842.5	116.0	10,381.0	3985.8
	Dry spruce	350.8	58.9	2846.9	396.9
Over-fire	Hard coal	2707.8	227.8	9039.6	4019.5
	Brown coal	3094.2	546.1	7003.7	781.9
	Brown coal briquettes	916.5	280.6	6238.2	1910.3
	Wet spruce	854.2	73.3	10,337.5	2426.9
	Dry spruce	485.3	204.7	9315.6	2532.5

## Data Availability

Dataset available on request from the authors.

## Supplementary Materials

The following supporting information can be downloaded at: https://www.mdpi.com/article/10.3390/toxics13080619/s1, Table S1. Combustion parameters; Table S2. Fuel parameters; Table S3. Emission and genotoxicity results; Table S4. *p*-Values.
